# Telepsychiatry: what clinicians need to know about digital mental healthcare

**DOI:** 10.1192/bja.2022.42

**Published:** 2023-07

**Authors:** Thomas J. Brunt, Oliver Gale-Grant

**Affiliations:** Psychiatry core trainee at South London and Maudsley NHS Foundation Trust, London, UK, with an interest in telehealth and the use of technology in healthcare.; Conducts research in the MRC Centre for Neurodevelopmental Disorders and Department of Forensic and Neurodevelopmental Science at King's College London, London, UK, focusing on computational modelling of brain development.

**Keywords:** Clinical governance, community mental health teams, history of psychiatry, outcome studies, randomised controlled trial

## Abstract

The COVID-19 pandemic has rapidly accelerated the use of online and remote mental healthcare provision. The immediate need to transform services has not allowed for thorough examination of the literature supporting remote delivery of psychiatric care. In this article we review the history of telepsychiatry, the rationale for continuing to offer services remotely and the limitations of psychiatry without in-person care. Focusing on randomised controlled trials we find that evidence for the efficacy of remotely delivered psychiatric care compared with in-person treatment is of low quality and limited scope but does not demonstrate clear superiority of one care delivery method over the other.

## LEARNING OBJECTIVES

After reading this article you will be able to:

- summarise the history of telepsychiatry
- summarise research evidence regarding the efficacy of telepsychiatry
- describe the limitations of telepsychiatry.

The theoretical foundation of a psychiatric interview is the mental state examination. Incorporating elements of descriptive phenomenology and elements of empathic observation, the clinician seeks to describe in a semi-structured way processes and behaviours that the patient themselves may or may not be aware of (Taylor 1967; Bell 1977). Since the mid-1990s the generally accepted format of this examination has divided an individual's mental state into six categories – appearance and behaviour, mood and affect, speech and language, thought process and content, cognition, and insight (Trzepacz 1993) – of which three rely on prolonged physical observation.

Conducting a thorough psychiatric examination remotely therefore appears challenging. Despite this, remote psychiatry clinics have existed for at least 50 years (Urness 2004; Shore 2015). More recently the COVID-19 pandemic has made adoption of telepsychiatry near universal – a survey by the American Psychiatric Association found that 98% of psychiatrists were seeing patients remotely in 2021 (American Psychiatric Association 2021). Against this background an understanding of the research evidence comparing the efficacy of telepsychiatry with in-person care, and the limitations of that evidence, is vital for practising clinicians.

## Defining telepsychiatry

Telepsychiatry is usually defined as the use of electronic communication and information technologies to provide or support clinical psychiatric care at a distance. It is occasionally subdivided into ‘synchronous’ and ‘asynchronous’ types, the former referring to systems in which the patient and clinician communicate directly and simultaneously (such as a video call) and the latter referring to systems in which they may communicate indirectly or at different times (Drago 2016).

## A brief history of telepsychiatry, 1970–2019

Initial attempts at establishing remotely delivered psychiatric care universally took place in the USA – a country with a large population living in rural areas. The first system for clinical use was established in the early 1970s when a two-way video system was installed between a teaching hospital and a smaller rural clinic in Nebraska, USA. Patients still went to the clinic, sat in a waiting-room and were shown into their consultation by a member of staff, who remained present during the session (Wittson 1972). This programme was a success and appreciated by the local population, and a number of similar programmes were subsequently developed, with staff usually present in a remote clinic to help the patient use the video equipment (Dwyer 1973; Murphy 1974; Dongier 1986).

Although psychiatry seemed a specialty naturally suited to telehealth (owing to the low requirements for physical examination of the patient) technological problems with early systems, including extremely slow data transfer times, made natural conversation impossible. By the mid-1980s the only telepsychiatry programmes active were funded by research grants and were not delivering routine clinical care (Preston 1992). There remained low interest in telepsychiatry for the next two decades – a 1997 review article found 18 studies on telepsychiatry. Of these, five were economic or feasibility evaluations and eight were cohort studies with no control group. The five studies available at that time comparing in-person psychiatry with telepsychiatry all had small samples, and three had no group randomisation. The authors concluded that ‘evidence currently available is insufficient to suggest its widespread implementation’ (Baer 1997).

The next decade saw a rapid rise in interest in telepsychiatry, driven in part by the commercial availability of videoconferencing systems (Monnier 2003). The feasibility of these systems for multiple aspects of mental healthcare was demonstrated, including child and adolescent services (Alessi 2002), geriatric services (Johnston 2001), cognitive–behavioural therapy (CBT) (Bouchard 2000; Cowain 2001) and neuropsychology (Schopp 2000). Although interest was growing, the efficacy of these methods compared with typical care was less clear: a 2005 meta-analysis found 14 studies which used standardised tools to directly compare diagnostic accuracy of in-person and remote psychiatry, with insufficient evidence to draw any firm conclusions (Hyler 2005). The chief limitation identified was a lack of homogeneity in patient groups studied – among the 14 studies are a cohort of adults with obsessive–compulsive disorder, two cohorts of geriatric nursing home residents, an adolescent cohort and a cohort of forensic in-patients. The authors did not, however, find any evidence that telepsychiatry was inferior to in-person care, a finding that was replicated in another review in 2010, which included 10 randomised controlled trials (RCTs) comparing outcomes following in-person and remote psychiatric interventions (García-Lizana 2010), albeit with the same limitation of very differing patient groups and interventions.

From 2010 onwards the rationale for telepsychiatry use began to evolve from the need to provide care to geographically remote areas to the need to provide care that patients find convenient (Pakyurek 2010). Several studies published between 2005 and 2013 demonstrated subjective patient preference for video interviews over face to face, especially among adolescents (Hilty 2013). Proliferation of services was widespread, but evidence of their efficacy compared with in-person clinics remained limited. A review in 2016 found eight studies directly comparing the efficacy of telepsychiatry with in-person care – seven found no difference in outcome and one (focusing on CBT in bulimia nervosa) found in-person care to be superior (Hubley 2016).

Models of telepsychiatry care evolved rapidly from 2010. Prior to this time telepsychiatry typically referred to either the use of a fixed videoconferencing system connecting a satellite clinic to a larger site, or the use of telephone calls to engage a patient in their own home. With the rise of internet and mobile phone availability, psychiatric care could now be delivered via text message, online chat rooms and video calls (Mermelstein 2017).

**FIG 1:**
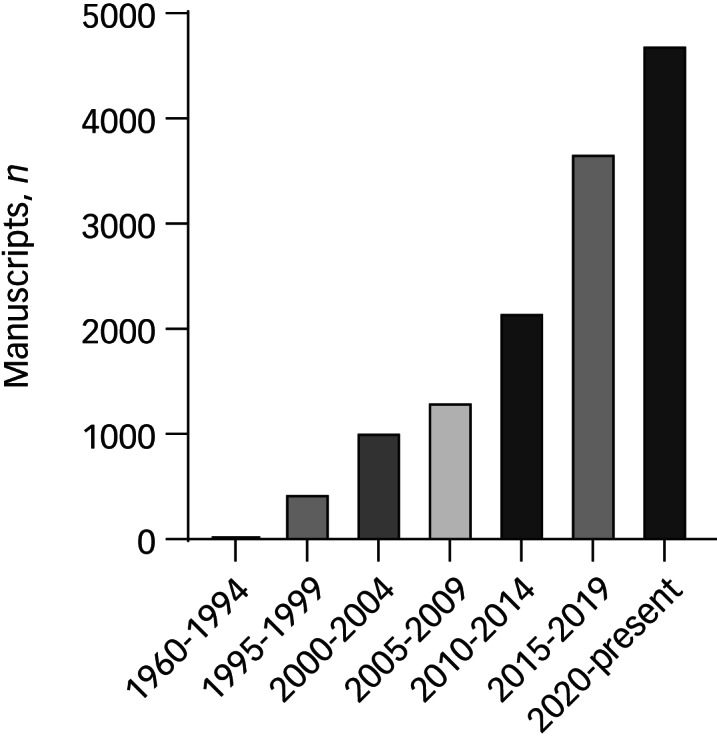
Number of published manuscripts containing the word ‘telepsychiatry’, 1960 to the present. Data retrieved from Google Scholar, February 2022.

Until the end of 2019 telepsychiatry services were still, however, largely delivered as short-term projects funded by research grants rather than substantial clinical services (Fig. 1).

## Telepsychiatry today

COVID-19 has transformed the practice of out-patient psychiatry in the UK and worldwide (Unützer 2020; Öngür 2020; Shore 2020). Substantial periods of social contact minimisation in many countries necessitated the rapid development of remotely delivered community care, and even as pandemic restrictions have eased, in many countries telepsychiatry remains the default mode of out-patient care (Mehrotra 2021; American Psychiatric Association 2021). Some professional bodies have released guidelines concerning the optimal use of telepsychiatry, but these are currently brief outline documents (the American Psychiatric Association's recommendations are just over two A4 sides in length for example, and the Royal College of Psychiatrists’ advice is a similar length) (RCPsych 2020; von Hafften 2021). Most are generally in agreement that telepsychiatry is viewed as safe and effective, and that clinicians are encouraged to offer appointments remotely if possible. In the UK the view that remote assessments are as robust as in-person ones was echoed by NHS England and the Department of Health and Social Care, who in 2020 endorsed remotely delivered Mental Health Act assessments. During the pandemic it was stated that ‘developments in digital technology are now such that staff may be satisfied, on the basis of video assessments, that they have personally seen or examined a person in a “suitable manner”’ (NHS England 2020), although notably this decision has since been overturned (*Devon Partnership NHS Trust v Secretary of State for Health and Social Care* [2021] EWHC 101). These changes were not unique to the UK – several other countries adjusted their legal frameworks to accommodate a rapid adoption of remote delivery of psychiatric care (Kinoshita 2020).

## Acceptability to patients

There is a large body of evidence studying patients’ perceptions, which in general demonstrates that patients are happy with remotely delivered care (Rohland 2000; Polinski 2016; Cowan 2020; Guinart 2020; Lal 2020). There is some emerging evidence to suggest that certain patient groups, such as female older adults, may favour in-person care (Christensen 2020) but more research is needed to draw firm conclusions in this regard. For a summary of current evidence of intergroup patterns of acceptability to patients see Barnett et al (2021).

Although studies agree that patients find telepsychiatry acceptable there is a key limitation to be aware of. The vast majority of patient opinion studies in telepsychiatry suffer extremely poor response rates: 22% in Guinart et al (2020), 33% in Rohland et al (2000) and 54% in Polinski et al (2016). There are clearly cohorts of individuals for whom telepsychiatry is not practical – people who do not own the necessary technology or are unfamiliar with it, people who struggle to adjust routines rapidly and people who lack a private space in which to talk freely, for example. Current qualitative methods that rely on voluntary participation are possibly failing to capture their perspectives.

## Acceptability to clinicians

There is limited evidence on the acceptability of telepsychiatry to clinicians, with most studies having small samples and the notable methodological flaw of only including clinicians who have already adopted telepsychiatry into their practice. Those that do exist largely demonstrate that clinicians find telepsychiatry systems easy to use and feel that their clinical judgement is not affected by seeing patients via a video screen (Elford 2001; Hensel 2020), although a minority report a clinician preference for in-person care (Hassan 2019). As ever in telepsychiatry, comparison between studies is limited by different patient groups, telepsychiatry systems and analysis methods. Elford et al studied a cohort of adolescent patients using a dedicated out-patient clinic video system, Hensel et al studied a system for remote emergency assessments in a general adult population and Hassan & Sharif focused on assessments of refugees. There is currently insufficient evidence to determine whether acceptability to clinicians is higher in certain circumstances (seeing familiar patients, for example) – for a review article discussing these questions in more detail see Hubley et al (2016).

## Diagnostic reliability

A small number of randomised trials have assessed the diagnostic reliability of a telepsychiatry assessment compared with an in-person assessment, all of which view an in-person diagnosis as their gold standard. Results of percentage agreement are reassuring: 69–82% in a child and adolescent sample (Brøndbo 2012), 75% in a random in-patient sample (Mazhari 2019), 76% in a general out-patient sample (Shore 2007), 92% in autism (Schutte 2015) and 96% in a child psychiatry sample (Elford 2000). There is a larger body of evidence of the reliability of structured tools being delivered remotely which shows that most commonly used tools are as effective via video call as they are in person – for a review see Drago et al (2016). A review focusing specifically on telephone assessments versus in-person assessments found an insufficient quality of literature to draw any conclusions (Muskens 2014).

## Therapeutic alliance

Therapeutic alliance, usually defined as the collaboration between patient and therapist to achieve a shared goal (typically recovery), is an important aspect of any psychiatric consultation. Body posture, eye contact and non-verbal communication (factors in building a positive alliance) are naturally affected by a remote care delivery method (Wootton 2003). A 2018 meta-analysis explicitly comparing measures of alliance in remote and in-person care (which focused exclusively on the delivery of formal therapy rather than general psychiatric assessments) included five studies and found that therapeutic alliance was marginally less strong in remote care, but that this did not translate to worse outcomes (Norwood 2018).

## Patient outcomes

A number of RCTs with samples over 100 have compared patient outcomes after telepsychiatry and in-person psychiatry (Table 1). Non-inferiority of telepsychiatry has been demonstrated in treating depression using medication, psychotherapy and psychoeducation (Ruskin 2004; Chong 2012; Moreno 2012; Egede 2015), although in two of these studies (which use the same patient population) individuals using telemedicine were prescribed antidepressants at a significantly higher rate (Chong 2012; Moreno 2012). One study demonstrated that structured therapy for post-traumatic stress disorder (PTSD) was more effective if delivered via video call than in person, although the telepsychiatry group received more overall care (Fortney 2015), and a second RCT of telepsychiatry versus in-person care in PTSD demonstrated no significant difference in outcome between the groups (Morland 2010). Two RCTs did not stratify by diagnosis but demonstrated non-inferiority of telepsychiatry (De Las Cuevas 2006; O'Reilly 2007). In one of these studies neither group of patients improved during the follow-up period, making the effectiveness of either intervention hard to judge (O'Reilly 2007). To our knowledge only one RCT (focusing on therapy for bulimia nervosa) to date has shown superior outcomes for in-person care compared with remote care, although differences were small and of dubious clinical significance (Mitchell 2008).

**TABLE 1:** Summary of randomised controlled trials (*n* > 100) directly comparing in-clinic or at-home telepsychiatry and in-person care

Study	Participants, *n*	Population; condition(s)	Type of care	Follow-up duration	Results
**Telepsychiatry delivered in the clinic**					
Ruskin et al, 2004	119	US Army veterans; depression	New assessments, psychoeducation, medication management, counselling	6 months	No difference in outcome
De Las Cuevas et al, 2006	140	General adults in the Canary Islands; any	New assessments, medication management, CBT	6 months	No difference in outcome
O'Reilly et al, 2007	495	General adults; any	New assessments, psychoeducation, medication, follow-up	4 months	No difference in outcome
Mitchell et al, 2008	128	General adults; bulimia nervosa	CBT	12 months	In-person therapy slightly more effective
Morland et al, 2010	125	US Army veterans; PTSD	Anger management therapy	6 months	No difference in outcome
Moreno et al, 2012	167	Hispanic adults; depression	New assessments, medication management, follow-up	6 months	No difference in outcome
Chong et al, 2012	167	Hispanic adults; depression	New assessments, medication management, follow-up	6 months	No difference in outcome
Egede et al, 2015	241	US Army veterans; depression	Behavioural activation therapy	12 months	No difference in outcome
Acierno et al, 2016	232	US Army veterans; PTSD or depression	Behavioural activation therapy	12 months	No difference in outcome
**Telepsychiatry delivered in the patient's home**					
Mohr et al, 2012	325	General adults; depression	Telephone-administered CBT	12 weeks	In-person therapy slightly more effective
Choi et al, 2014	158	Older adults; depression	Problem-solving therapy (via Skype video call)	6 months	No difference in outcome
Luxton et al, 2016	121	US Army veterans; depression	Behavioural activation therapy	3 months	In-person therapy slightly more effective
Acierno et al, 2017	132	US Army veterans; PTSD	Prolonged exposure therapy	6 months	In-person therapy slightly more effective
Glassman et al, 2019	125	US Arm veterans; PTSD	Cognitive processing therapy	6 months	No difference in outcome

PTSD, post-traumatic stress disorder; CBT, cognitive–behavioural therapy.

Three further RCTs use adjunctive care designs, comparing a group receiving treatment as usual with a group additionally receiving a telepsychiatry intervention (Fortney 2013; Myers 2015; Hulsbosch 2016). All show that the group receiving adjunctive care had better outcomes. One used a complex design in which one group's direct care providers received expert advice remotely (Fortney 2013). Previous reviewers have expressed concern as to the methodological quality of adjunctive telepsychiatry research. A 2018 review included eight studies, of which five were judged to be of low quality and three of adequate quality. All five low-quality studies found that the technological adjunct reduced remission rates, whereas none of the adequate-quality studies did (Koblauch 2018).

An extremely important limitation of the RCTs above is that they all use a model of care in which the ‘telepsychiatry’ intervention was delivered in a clinic – the patient experience is identical to a typical out-patient appointment other than the psychiatrist is replaced by a video screen. This is obviously quite different from the manner in which telepsychiatry has been delivered during the COVID-19 pandemic. There are a small number of studies comparing in-person care with care delivered via video with the patient at home. Two studies focusing on PTSD found generally similar results between groups, with slightly improved 3-month follow-up scores in the in-person group (Acierno 2016, 2017). This slight preference for in-person therapy is also reflected in one study comparing at-home versus in-person treatment for depression which showed better outcomes in the in-person care group (Luxton 2016) and one showing better outcomes for in-person therapy for PTSD (Glassman 2019). Conversely, however, one RCT comparing at-home telepsychiatry with in-person clinic appointments found no difference between groups (Choi 2014). Finally, one study compared depression care via telephone with in-person clinic care, finding in-person care slightly superior (Mohr 2012). One limitation in comparing in-clinic and at-home studies is that the available at-home RCTs exclusively examine formal therapies, whereas in-clinic studies examine a broader range of care modalities, including psychiatric assessments of new patients, medication management and psychoeducation sessions. RCTs of in-clinic therapies universally show no outcome difference, so it is reasonable to hypothesise that setting, not just the type of care delivered, is important in outcome prediction.

Research discussed so far concerns live appointments delivered remotely. There is also a significant body of literature examining ‘asynchronous telepsychiatry’ – i.e. care delivered by messaging systems, chat rooms or other modalities in which the clinician and patient are not present at the same time. Discussing this in detail is outside the scope of this article – for a recent review see O'Keefe et al (2019).

## Limitations of telepsychiatry

Three chief barriers to adoption of telepsychiatry are frequently discussed in the literature.

### Emergency care

Remote management of psychiatric emergencies is essentially an untested field. A 2019 review concluded that current evidence does not allow any conclusions to be drawn as to the suitability of remote crisis assessments (Reinhardt 2019). Feasibility studies of emergency telepsychiatry primarily use the care model popular pre-1990 of having one clinician physically present in the room with the patient and a second (typically more specialised) clinician also assessing via video screen, and in general report that clinician decision-making is the same regardless of whether the assessment is performed remotely or in person (Seidel 2014; Roberts 2017; Freeman 2020).

### Data, privacy and governance concerns

The storage of confidential data is governed by legislative frameworks in most countries, and prior to the pandemic few of these had adequate provision for the mass use of videoconferencing. The solution adopted has in general been a temporary relaxation of medicolegal constraints (Kinoshita 2020), allowing providers to use common applications such as WhatsApp and Skype to perform interviews. This will inevitably have led to confidential information being stored on third-party servers, and in the longer term solutions to this issue are needed. A second related problem is that the geographical location of the patient and clinician may no longer be the same if care is delivered via video call – medical licences are usually national (or, in the USA, regional) and it is currently unclear whether the physical location of the clinician or patient is important in determining the limit of practice. These problems have been apparent for some time and no widely adopted solutions are yet forthcoming (Baker 2011).

### Inaccessibility for certain patients

Communicating via video screen is a familiar part of life for many, but even after the pandemic some people are yet to use this technology. One study has attempted to quantify this problem, comparing ‘conversion rates’ from in-person to remote appointments during COVID in an out-patient clinic. Perhaps surprisingly it found that severity of mental illness did not predict uptake of telepsychiatry, but age did, with older patients far less likely to make the transition from in-person appointments (Miu 2021).

## Discussion and recommendations for practice

Drawing firm conclusions regarding the efficacy, safety or tolerability of telepsychiatry compared with in-person care is extremely difficult. Problems with existing evidence make generalisable conclusions impossible.

Most RCTs use clearly defined patient populations, such as individuals with depression and good social functioning (Chong 2012; Fortney 2013). To our knowledge, there is no good-quality RCT investigating whether telepsychiatry is effective in anxiety, psychosis or personality disorders, for example (although some small RCTs showed promising results in anxiety – for a review see Berryhill et al (2018)). A second key limitation is that the majority of RCTs currently published are investigating a model of care in which patients travel to a clinic where they are seen by a clinician via a video screen (Table 1). In one study the psychiatrist delivering telepsychiatry was physically located in the same building as the patient (Ruskin 2004). This is more similar to an in-person consultation than a consultation via smartphone – the patient will still travel to the clinic, will interact with other members of staff and, in some cases, will even have a secondary clinician present during their telepsychiatry appointment (Morland 2010). This is not to say that at-home telepsychiatry care cannot be effective – one RCT in the field of sleep medicine comparing a CBT intervention for insomnia via at-home video call with the same intervention in person found no difference in outcome between groups (Arnedt 2020).

The multiple possible formats of a telepsychiatry delivery (a video screen in clinic, telephone call, video chat, dedicated web apps, for example) are occasionally compared in RCTs. Summarising this literature is outside the scope of this review. However, we can use these studies to further hypothesise about the efficacy of at-home telepsychiatry compared with in-clinic in-person psychiatry. One RCT (with 73 participants) has shown that an at-home online delivery of care was as effective as an in-clinic video screen for the delivery of CBT in insomnia (Holmqvist 2014). There are also a number of observational studies that suggest that telephone-based at-home care can be effective (although without comparison with in-person care) (Varker 2019).

Studies of diagnostic reliability via remote assessment are reassuring, often showing the percentage agreement on diagnosis between a clinician assessing in person and virtually at above 75%. This is comparable to the likelihood of agreement between two psychiatrists assessing in person (Aboraya 2006). Also reassuring are studies of patients’ opinions of telepsychiatry, which are in general positive (Sharma 2021), although studies must be interpreted in the context of low response rates and only receiving information from patients who have used telepsychiatry. A more cautious approach to this part of the literature would be to conclude that telepsychiatry is readily accepted by some patients.

Despite the lack of good-quality RCTs supporting the efficacy of telepsychiatry it is important to note that there is equally not a lot of evidence of in-person care being superior to telepsychiatry. To date only one RCT, using an in-clinic videoconferencing system, has reported this finding (Mitchell 2008) and three have demonstrated in-person care to be slightly superior to telepsychiatry delivered to the patient at home (Mohr 2012; Acierno 2016; Luxton 2016). The magnitude of intergroup difference is small in all these studies. It is reasonable to hypothesise that at-home telepsychiatry is as effective as in-person care for at least a subgroup of patients.

There are equally groups of individuals for whom it is reasonable to hypothesise that telepsychiatry may not be effective. One such group is individuals with long-term complex needs. A recent narrative review comparing telepsychiatry and in-person care for the long-term management of individuals with multiple comorbidities (not exclusively focused on psychiatry) suggested that for individuals with complex needs in-person care is superior to telehealth methods (Béland 2021), and a recent review of telepsychiatry during the COVID-19 pandemic suggested that individuals with psychosis, autism and intellectual difficulties in particular struggled to adapt to remote appointments (Appleton 2021). In general, however, there is not a sufficient body of evidence to decide firmly whether certain groups are not suited to remotely delivered care – for a recent review examining patient opinion and acceptability differences between groups see Barnett et al (2021).

We have focused primarily on RCTs in this review. There does exist, however, a significant body of literature using other study designs to assess the efficacy or suitability of telepsychiatry services. Although much of this literature is limited by methodological constraints, it is worth noting that there is at least preliminary evidence to suggest that telepsychiatry can be effective in a wide range of patient groups, including general adult (Coughtrey 2016), older adult (Harerimana 2019) and substance misuse services (Lin 2019).

One further aspect of the adoption of telepsychiatry that we have not addressed in this review is the health economics argument for it. Telepsychiatry is in general thought to be cheaper for the health system to provide than in-person psychiatry, and has additional advantages to the patient in saving time and money travelling to clinics (Naslund 2020).

### Recommendations

Given the above, what is a sensible and scientifically robust way to approach telepsychiatry (Box 1)? The first and most important point is to allow the patient to choose which way they prefer to access care. The second point is to be realistic that it is unknown how effective telepsychiatry may be in several diagnostic groups and that for these patients a blended approach is probably more sensible. It is equally unknown how effective the specific style of care that has become widespread during the COVID-19 pandemic (i.e. unstructured telephone or video interviews with patients who are in their own homes) may be. The limited RCT evidence addressing this specific question (Table 1) suggests that it may be slightly less effective than in-person care.

BOX 1 Recommendations for implementing telepsychiatry

- Be aware that there is limited or no evidence for the efficacy of remotely delivered psychiatric care compared with in-person care in most circumstances
- Be mindful that remotely delivered care will not suit all patients, although there will likely be some for whom it is preferable – allow the patient to make this choice if possible
- Diagnostic reliability does not appear to be altered by remote assessment – telepsychiatry is not more or less well evidenced in certain settings
- Complex case management is unlikely to be as effectively performed via telepsychiatry as via in-person care

There is also limited evidence regarding which aspects of psychiatric care may and may not be feasible remotely. RCTs usually focus on one intervention, such as a structured therapy programme (e.g. Luxton 2016) or psychiatrist-delivered medication management (e.g. O'Reilly 2007). There is not yet sufficient evidence to decide whether certain activities are more appropriately delivered remotely than others, although one common finding is that formal structured assessments are as robust remotely as in person (Drago 2016). There are some critical services, such as community crisis teams, about which there is no current evidence examining their feasibility as telepsychiatry services (Barnett 2021).

It is important to balance the potential limitations of telepsychiatry against its potential benefits to the healthcare system – a community psychiatrist may be able to see more patients in a day without travelling between them, access to psychology and other services may be greater virtually than in person, and the overall cost of telepsychiatry may be lower than the cost of delivering the same care in person. There are also regional shortages of psychiatrists in the UK and many countries worldwide – telepsychiatry is a very promising route to resolving these geographical inequalities.

The rapid introduction of telepsychiatry could be seen as a challenge to prevailing beliefs about psychiatric interview and the value of mental state examination. A number of things traditionally taught as of paramount importance in assessing mental state (observing the patient's outfit, their belongings and movement of limbs during the interview, for example) are very difficult to do via video call and impossible to do via telephone (Stringer 2020). The fact that omitting or substantially reducing these aspects of the mental state examination appears to have limited effect on diagnostic accuracy, therapeutic alliance (Reese 2016) and patient outcome should be of interest.

## Conclusions

Telepsychiatry is very likely to be a part of out-patient psychiatry for the foreseeable future – at the time of writing, several National Health Service trusts are advertising fully remote consultant psychiatrist posts. This is the greatest change to the manner in which psychiatry is practised in the past 70 years, but the evidence base underlying this change is severely limited. High-quality RCTs are urgently needed to assess the safety, efficacy and long-term tolerability of remotely delivered care in a number of common conditions – a conclusion that is commonly reached in systematic reviews (Drago 2016; Koblauch 2018; Sales 2018; Zhao 2021). Until such studies are available, offering a blended approach to out-patient care with both in-person and virtual appointments as per patient preference appears to be prudent.

## Supplementary material

For supplementary material accompanying this paper visit https://doi.org/10.1192/bja.2022.42. click here to view supplementary material

## MCQs

Select the single best option for each question stem

1. For which of the following conditions has an RCT demonstrated non-inferiority of remotely delivered versus in-person care?
  - Depression
  - Bipolar disorder
  - Schizophrenia
  - Emotionally unstable personality disorder
  - All of the above.
2. Which of the following telepsychiatry therapy modalities has been shown via RCT to be non-inferior to in-person therapy for the treatment of depression?
  - Mindfulness
  - Cognitive analytic therapy
  - CBT
  - Psychodynamic therapy
  - All of the above.
3. Which of the following is considered a telepsychiatry intervention?
  - Video call between patient and psychiatrist
  - Video call between members of the clinical team
  - Using an online self-help resource
  - Text chat with a support worker
  - All of the above.
4. Which of the following groups has telepsychiatry been shown to be less readily adopted by?
  - Elderly patients
  - Individuals with severe mental illness
  - Individuals with intellectual disability
  - Individuals not being treated in their first language
  - All of the above.
5. Which of the following modes of telepsychiatry delivery has been shown to be non-inferior to in-person care in at least one RCT?
  - App-based delivery
  - Guided online self-help
  - In-clinic video call
  - Text chat
  - All of the above.

MCQ answers 1 a 2 c 3 e 4 a 5 c
