# Supplementary material for: Telepsychiatry: what clinicians need to know about digital mental healthcare
Source: BJPsych Adv. 2023 Jul;29(4):230–8. doi: 10.1192/bja.2022.42 (PMC10374879; doi:10.1192/bja.2022.42)
Supplement: Supplementary file 1 [file S2056467822000421sup001.docx]

**Supplementary Material for**

**Telepsychiatry: what clinicians need to know about digital mental health**

Thomas J Brunt^1^, Oliver Gale-Grant^2,3^

1. South London and Maudsley NHS Foundation Trust
2. MRC Centre for Neurodevelopmental Disorders, King’s College London
3. Department of Forensic and Neurodevelopmental Science, King’s College London

**Summary**

The Covid-19 pandemic has rapidly accelerated the use of online and remote mental health care provision. The immediate need to transform services has not allowed for thorough examination of the literature supporting remote delivery of psychiatric care. In this article we review the history of telepsychiatry, the rationale for continuing to offer services remotely and the limitations of psychiatry without in person care. Focusing on randomised controlled trials we find that evidence for the efficacy of remotely delivered psychiatric care compared to in person treatment is of low quality and limited scope but does not demonstrate clear superiority of one care delivery method over the other.

**References**

ABORAYA, A., RANKIN, E., FRANCE, C., EL-MISSIRY, A. & JOHN, C. 2006. The reliability of psychiatric diagnosis revisited: The clinician's guide to improve the reliability of psychiatric diagnosis. *Psychiatry (Edgmont),* 3**,** 41.

ACIERNO, R., GROS, D. F., RUGGIERO, K. J., HERNANDEZ-TEJADA, B. M., KNAPP, R. G., LEJUEZ, C. W., MUZZY, W., FRUEH, C. B., EGEDE, L. E. & TUERK, P. W. 2016. BEHAVIORAL ACTIVATION AND THERAPEUTIC EXPOSURE FOR POSTTRAUMATIC STRESS DISORDER: A NONINFERIORITY TRIAL OF TREATMENT DELIVERED IN PERSON VERSUS HOME-BASED TELEHEALTH. *Depress Anxiety,* 33**,** 415-23.

ACIERNO, R., KNAPP, R., TUERK, P., GILMORE, A. K., LEJUEZ, C., RUGGIERO, K., MUZZY, W., EGEDE, L., HERNANDEZ-TEJADA, M. A. & FOA, E. B. 2017. A non-inferiority trial of Prolonged Exposure for posttraumatic stress disorder: In person versus home-based telehealth. *Behaviour research and therapy,* 89**,** 57-65.

ALESSI, N. E. 2002. Telepsychiatric care for a depressed adolescent. *Journal of the American Academy of Child & Adolescent Psychiatry,* 8**,** 894-895.

APA. 2021. Psychiatrists Use of Telepsychiatry During COVID-19 Public Health Emergency. [Accessed 08/02/2022 2022].

APPLETON, R., WILLIAMS, J., VERA SAN JUAN, N., NEEDLE, J. J., SCHLIEF, M., JORDAN, H., SHERIDAN RAINS, L., GOULDING, L., BADHAN, M., ROXBURGH, E., BARNETT, P., SPYRIDONIDIS, S., TOMASKOVA, M., MO, J., HARJU-SEPPÄNEN, J., HAIME, Z., CASETTA, C., PAPAMICHAIL, A., LLOYD-EVANS, B., SIMPSON, A., SEVDALIS, N., GAUGHRAN, F. & JOHNSON, S. 2021. Implementation, Adoption, and Perceptions of Telemental Health During the COVID-19 Pandemic: Systematic Review. *Journal of medical Internet research,* 23**,** e31746-e31746.

ARNEDT, J. T., CONROY, D. A., MOONEY, A., FURGAL, A., SEN, A. & EISENBERG, D. 2020. Telemedicine versus face-to-face delivery of cognitive behavioral therapy for insomnia: a randomized controlled noninferiority trial. *Sleep,* 44.

BAER, L., ELFORD, D. R. & CUKOR, P. 1997. Telepsychiatry at Forty: What Have We Learned? *Harvard Review of Psychiatry,* 5**,** 7-17.

BAKER, D. C. & BUFKA, L. F. 2011. Preparing for the telehealth world: Navigating legal, regulatory, reimbursement, and ethical issues in an electronic age. *Professional Psychology: Research and Practice,* 42**,** 405.

BARNETT, P., GOULDING, L., CASETTA, C., JORDAN, H., SHERIDAN-RAINS, L., STEARE, T., WILLIAMS, J., WOOD, L., GAUGHRAN, F. & JOHNSON, S. 2021. Implementation of Telemental Health Services Before COVID-19: Rapid Umbrella Review of Systematic Reviews. *Journal of medical Internet research,* 23**,** e26492-e26492.

BÉLAND, S., DUMONT-SAMSON, O. & HUDON, C. 2021. Case Management and Telehealth: A Scoping Review. *Telemedicine and e-Health,* 28**,** 11-23.

BELL, R. & HALL, R. C. 1977. The mental status examination. *Am Fam Physician,* 16**,** 145-52.

BERRYHILL, M. B., HALLI-TIERNEY, A., CULMER, N., WILLIAMS, N., BETANCOURT, A., KING, M. & RUGGLES, H. 2018. Videoconferencing psychological therapy and anxiety: a systematic review. *Family Practice,* 36**,** 53-63.

BOUCHARD, S., PAYEUR, R., RIVARD, V., ALLARD, M., PAQUIN, B., RENAUD, P. & GOYER, L. 2000. Cognitive behavior therapy for panic disorder with agoraphobia in videoconference: Preliminary results. *CyberPsychology & Behavior,* 3**,** 999-1007.

BRØNDBO, H., MATHIASSEN, B., MARTINUSSEN, M., HEIERVANG, E., ERIKSEN, M. & KVERNMO, S. 2012. Agreement on Web-based Diagnoses and Severity of Mental Health Problems in Norwegian Child and Adolescent Mental Health Services. *Clinical practice and epidemiology in mental health : CP & EMH,* 8**,** 16-21.

CHOI, N. G., MARTI, C. N., BRUCE, M. L., HEGEL, M. T., WILSON, N. L. & KUNIK, M. E. 2014. Six-month postintervention depression and disability outcomes of in-home telehealth problem-solving therapy for depressed, low-income homebound older adults. *Depress Anxiety,* 31**,** 653-61.

CHONG, J. & MORENO, F. 2012. Feasibility and acceptability of clinic-based telepsychiatry for low-income Hispanic primary care patients. *Telemed J E Health,* 18**,** 297-304.

CHRISTENSEN, L. F., MOLLER, A. M., HANSEN, J. P., NIELSEN, C. T. & GILDBERG, F. A. 2020. Patients' and providers' experiences with video consultations used in the treatment of older patients with unipolar depression: A systematic review. *J Psychiatr Ment Health Nurs,* 27**,** 258-271.

COUGHTREY, A. E. & PISTRANG, N. 2016. The effectiveness of telephone-delivered psychological therapies for depression and anxiety: A systematic review. *Journal of Telemedicine and Telecare,* 24**,** 65-74.

COWAIN, T. 2001. Cognitive–behavioural therapy via videoconferencing to a rural area. *Australian & New Zealand Journal of Psychiatry,* 35**,** 62-64.

COWAN, A., JOHNSON, R. & CLOSE, H. 2020. Telepsychiatry in psychotherapy practice. *Innovations in clinical neuroscience,* 17**,** 23.

DE LAS CUEVAS, C., ARREDONDO, M. T., CABRERA, M. F., SULZENBACHER, H. & MEISE, U. 2006. Randomized clinical trial of telepsychiatry through videoconference versus face-to-face conventional psychiatric treatment. *Telemed J E Health,* 12**,** 341-50.

DONGIER, M., TEMPIER, R., LALINEC-MICHAUD, M. & MEUNIER, D. 1986. Telepsychiatry: psychiatric consultation through two-way television. A controlled study. *Can J Psychiatry,* 31**,** 32-4.

DRAGO, A., WINDING, T. N. & ANTYPA, N. 2016. Videoconferencing in psychiatry, a meta-analysis of assessment and treatment. *European Psychiatry,* 36**,** 29-37.

DWYER, T. F. 1973. Telepsychiatry: psychiatric consultation by interactive television. *Am J Psychiatry,* 130**,** 865-9.

EGEDE, L. E., ACIERNO, R., KNAPP, R. G., LEJUEZ, C., HERNANDEZ-TEJADA, M., PAYNE, E. H. & FRUEH, B. C. 2015. Psychotherapy for depression in older veterans via telemedicine: a randomised, open-label, non-inferiority trial. *Lancet Psychiatry,* 2**,** 693-701.

ELFORD, D. R., WHITE, H., ST JOHN, K., MADDIGAN, B., GHANDI, M. & BOWERING, R. 2001. A prospective satisfaction study and cost analysis of a pilot child telepsychiatry service in Newfoundland. *J Telemed Telecare,* 7**,** 73-81.

ELFORD, R., WHITE, H., BOWERING, R., GHANDI, A., MADDIGGAN, B., ST JOHN, K., HOUSE, M., HARNETT, J., WEST, R. & BATTCOCK, A. 2000. A randomized, controlled trial of child psychiatric assessments conducted using videoconferencing. *J Telemed Telecare,* 6**,** 73-82.

ENGLAND AND WALES HIGH COURT 2021. Devon Partnership NHS Trust v Secretary of State for Health and Social Care [2021] EWHC 101. London: Royal Courts of Justice.

FORTNEY, J. C., PYNE, J. M., KIMBRELL, T. A., HUDSON, T. J., ROBINSON, D. E., SCHNEIDER, R., MOORE, W. M., CUSTER, P. J., GRUBBS, K. M. & SCHNURR, P. P. 2015. Telemedicine-Based Collaborative Care for Posttraumatic Stress Disorder: A Randomized Clinical Trial. *JAMA Psychiatry,* 72**,** 58-67.

FORTNEY, J. C., PYNE, J. M., MOUDEN, S. B., MITTAL, D., HUDSON, T. J., SCHROEDER, G. W., WILLIAMS, D. K., BYNUM, C. A., MATTOX, R. & ROST, K. M. 2013a. Practice-based versus telemedicine-based collaborative care for depression in rural federally qualified health centers: a pragmatic randomized comparative effectiveness trial. *Am J Psychiatry,* 170**,** 414-25.

FORTNEY, J. C., PYNE, J. M., MOUDEN, S. B., MITTAL, D., HUDSON, T. J., SCHROEDER, G. W., WILLIAMS, D. K., BYNUM, C. A., MATTOX, R. & ROST, K. M. 2013b. Practice-based versus telemedicine-based collaborative care for depression in rural federally qualified health centers: a pragmatic randomized comparative effectiveness trial. *The American journal of psychiatry,* 170**,** 414-425.

FREEMAN, R. E., BOGGS, K. M., ZACHRISON, K. S., FREID, R. D., SULLIVAN, A. F., ESPINOLA, J. A. & CAMARGO JR, C. A. 2020. National study of telepsychiatry use in US emergency departments. *Psychiatric services,* 71**,** 540-546.

GARCÍA-LIZANA, F. & MUÑOZ-MAYORGA, I. 2010. What about telepsychiatry? A systematic review. *The Primary Care Companion for CNS Disorders,* 12**,** 26919.

GLASSMAN, L. H., MACKINTOSH, M. A., TALKOVSKY, A., WELLS, S. Y., WALTER, K. H., WICKRAMASINGHE, I. & MORLAND, L. A. 2019. Quality of life following treatment for PTSD: Comparison of videoconferencing and in-person modalities. *J Telemed Telecare,* 25**,** 123-127.

GUINART, D., MARCY, P., HAUSER, M., DWYER, M. & KANE, J. M. 2020. Patient Attitudes Toward Telepsychiatry During the COVID-19 Pandemic: A Nationwide, Multisite Survey. *JMIR Ment Health,* 7**,** e24761.

HARERIMANA, B., FORCHUK, C. & O'REGAN, T. 2019. The use of technology for mental healthcare delivery among older adults with depressive symptoms: A systematic literature review. *Int J Ment Health Nurs,* 28**,** 657-670.

HASSAN, A. & SHARIF, K. 2019. Efficacy of Telepsychiatry in Refugee Populations: A Systematic Review of the Evidence. *Cureus,* 11**,** e3984.

HENSEL, J., GRAHAM, R., ISAAK, C., AHMED, N., SAREEN, J. & BOLTON, J. 2020. A Novel Emergency Telepsychiatry Program in a Canadian Urban Setting: Identifying and Addressing Perceived Barriers for Successful Implementation: Un nouveau programme de télépsychiatrie d’urgence en milieu urbain canadien: Identifier et aborder les obstacles perçus d’une mise en œuvre réussie. *The Canadian Journal of Psychiatry,* 65**,** 559-567.

HILTY, D. M., FERRER, D. C., PARISH, M. B., JOHNSTON, B., CALLAHAN, E. J. & YELLOWLEES, P. M. 2013. The Effectiveness of Telemental Health: A 2013 Review. *Telemedicine and e-Health,* 19**,** 444-454.

HOLMQVIST, M., VINCENT, N. & WALSH, K. 2014. Web- vs telehealth-based delivery of cognitive behavioral therapy for insomnia: a randomized controlled trial. *Sleep Medicine,* 15**,** 187-195.

HUBLEY, S., LYNCH, S. B., SCHNECK, C., THOMAS, M. & SHORE, J. 2016. Review of key telepsychiatry outcomes. *World journal of psychiatry,* 6**,** 269-282.

HULSBOSCH, A. M., NUGTER, M. A., TAMIS, P. & KROON, H. 2016. Videoconferencing in a mental health service in The Netherlands: A randomized controlled trial on patient satisfaction and clinical outcomes for outpatients with severe mental illness. *Journal of Telemedicine and Telecare,* 23**,** 513-520.

HYLER, S. E., GANGURE, D. P. & BATCHELDER, S. T. 2005. Can Telepsychiatry Replace In-Person Psychiatric Assessments? A Review and Meta-Analysis of Comparison Studies. *CNS Spectrums,* 10**,** 403-415.

JOHNSTON, D. & JONES III, B. N. 2001. Telepsychiatry consultations to a rural nursing facility: a 2-year experience. *Journal of Geriatric Psychiatry and Neurology,* 14**,** 72-75.

KINOSHITA, S., CORTRIGHT, K., CRAWFORD, A., MIZUNO, Y., YOSHIDA, K., HILTY, D., GUINART, D., TOROUS, J., CORRELL, C. U., CASTLE, D. J., ROCHA, D., YANG, Y., XIANG, Y.-T., KØLBÆK, P., DINES, D., ELSHAMI, M., JAIN, P., KALLIVAYALIL, R., SOLMI, M., FAVARO, A., VERONESE, N., SEEDAT, S., SHIN, S., SALAZAR DE PABLO, G., CHANG, C.-H., SU, K.-P., KARAS, H., KANE, J. M., YELLOWLEES, P. & KISHIMOTO, T. 2020. Changes in telepsychiatry regulations during the COVID-19 pandemic: 17 countries and regions' approaches to an evolving healthcare landscape. *Psychological Medicine***,** 1-8.

KOBLAUCH, H., REINHARDT, S. M., LISSAU, W. & JENSEN, P.-L. 2018. The effect of telepsychiatric modalities on reduction of readmissions in psychiatric settings: A systematic review. *Journal of telemedicine and telecare,* 24**,** 31-36.

LAL, S., ABDEL-BAKI, A., SUJANANI, S., BOURBEAU, F., SAHED, I. & WHITEHEAD, J. 2020. Perspectives of Young Adults on Receiving Telepsychiatry Services in an Urban Early Intervention Program for First-Episode Psychosis: A Cross-Sectional, Descriptive Survey Study. *Frontiers in Psychiatry,* 11.

LIN, L., CASTEEL, D., SHIGEKAWA, E., WEYRICH, M. S., ROBY, D. H. & MCMENAMIN, S. B. 2019. Telemedicine-delivered treatment interventions for substance use disorders: A systematic review. *Journal of Substance Abuse Treatment,* 101**,** 38-49.

LUXTON, D. D., PRUITT, L. D., WAGNER, A., SMOLENSKI, D. J., JENKINS-GUARNIERI, M. A. & GAHM, G. 2016. Home-based telebehavioral health for U.S. military personnel and veterans with depression: A randomized controlled trial. *J Consult Clin Psychol,* 84**,** 923-934.

MAZHARI, S., GHAFFARI NEJAD, A., MOFAKHAMI, O., RAAII, F. & BAHAADINBEIGY, K. 2019. Evaluating the Diagnostic Agreement between Telepsychiatry Assessment and Face-to-Face Visit: A Preliminary Study. *Iran J Psychiatry,* 14**,** 236-241.

MEHROTRA, A., CHERNEW, M., LINETSKY, D., HATCH, H., CUTLER, D. & SCHNEIDER, E. C. 2021. The impact of COVID-19 on outpatient visits in 2020: visits remained stable, despite a late surge in cases. *New York: Commonwealth Fund*.

MERMELSTEIN, H., GUZMAN, E., RABINOWITZ, T., KRUPINSKI, E. & HILTY, D. 2017. The Application of Technology to Health: The Evolution of Telephone to Telemedicine and Telepsychiatry: A Historical Review and Look at Human Factors. *Journal of Technology in Behavioral Science,* 2**,** 5-20.

MITCHELL, J. E., CROSBY, R. D., WONDERLICH, S. A., CROW, S., LANCASTER, K., SIMONICH, H., SWAN-KREMEIER, L., LYSNE, C. & MYERS, T. C. 2008. A randomized trial comparing the efficacy of cognitive-behavioral therapy for bulimia nervosa delivered via telemedicine versus face-to-face. *Behav Res Ther,* 46**,** 581-92.

MIU, A. S., VO, H. T., PALKA, J. M., GLOWACKI, C. R. & ROBINSON, R. J. 2021. Teletherapy with serious mental illness populations during COVID-19: telehealth conversion and engagement. *Counselling Psychology Quarterly,* 34**,** 704-721.

MOHR, D. C., HO, J., DUFFECY, J., REIFLER, D., SOKOL, L., BURNS, M. N., JIN, L. & SIDDIQUE, J. 2012. Effect of telephone-administered vs face-to-face cognitive behavioral therapy on adherence to therapy and depression outcomes among primary care patients: a randomized trial. *Jama,* 307**,** 2278-85.

MONNIER, J., KNAPP, R. G. & FRUEH, B. C. 2003. Recent Advances in Telepsychiatry: An Updated Review. *Psychiatric Services,* 54**,** 1604-1609.

MORENO, F. A., CHONG, J., DUMBAULD, J., HUMKE, M. & BYREDDY, S. 2012. Use of standard Webcam and Internet equipment for telepsychiatry treatment of depression among underserved Hispanics. *Psychiatr Serv,* 63**,** 1213-7.

MORLAND, L. A., GREENE, C. J., ROSEN, C. S., FOY, D., REILLY, P., SHORE, J., HE, Q. & FRUEH, B. C. 2010. Telemedicine for anger management therapy in a rural population of combat veterans with posttraumatic stress disorder: a randomized noninferiority trial. *J Clin Psychiatry,* 71**,** 855-63.

MURPHY JR, R. & BIRD, K. T. 1974. Telediagnosis: a new community health resource. Observations on the feasibility of telediagnosis based on 1000 patient transactions. *American Journal of Public Health,* 64**,** 113-119.

MUSKENS, E. M., LUCASSEN, P., GROENLEER, W., VAN WEEL, C., OUDE VOSHAAR, R. & SPECKENS, A. 2014. Psychiatric diagnosis by telephone: is it an opportunity? *Soc Psychiatry Psychiatr Epidemiol,* 49**,** 1677-89.

MYERS, K., VANDER STOEP, A., ZHOU, C., MCCARTY, C. A. & KATON, W. 2015. Effectiveness of a telehealth service delivery model for treating attention-deficit/hyperactivity disorder: a community-based randomized controlled trial. *J Am Acad Child Adolesc Psychiatry,* 54**,** 263-74.

NASLUND, J. A., MITCHELL, L. M., JOSHI, U., NAGDA, D. & LU, C. 2020. Economic evaluation and costs of telepsychiatry programmes: A systematic review. *Journal of telemedicine and telecare***,** 1357633X20938919.

NHS ENGLAND 2020. Legal guidance for mental health, learning disability and autism, and specialised commissioning services supporting people of all ages during the coronavirus pandemic 30 March 2020, Version 1.

NORWOOD, C., MOGHADDAM, N. G., MALINS, S. & SABIN-FARRELL, R. 2018. Working alliance and outcome effectiveness in videoconferencing psychotherapy: A systematic review and noninferiority meta-analysis. *Clinical Psychology & Psychotherapy,* 25**,** 797-808.

O'REILLY, R., BISHOP, J., MADDOX, K., HUTCHINSON, L., FISMAN, M. & TAKHAR, J. 2007. Is Telepsychiatry Equivalent to Face-to-Face Psychiatry? Results From a Randomized Controlled Equivalence Trial. *Psychiatric Services,* 58**,** 836-843.

O’KEEFE, M., WHITE, K. & JENNINGS, J. A. C. 2019. Asynchronous telepsychiatry: A systematic review. *Journal of Telemedicine and Telecare,* 27**,** 137-145.

ÖNGÜR, D., PERLIS, R. & GOFF, D. 2020. Psychiatry and COVID-19. *JAMA,* 324**,** 1149-1150.

PAKYUREK, M., YELLOWLEES, P. & HILTY, D. 2010. The Child and Adolescent Telepsychiatry Consultation: Can It Be a More Effective Clinical Process for Certain Patients Than Conventional Practice? *Telemedicine and e-Health,* 16**,** 289-292.

POLINSKI, J. M., BARKER, T., GAGLIANO, N., SUSSMAN, A., BRENNAN, T. A. & SHRANK, W. H. 2016. Patients’ Satisfaction with and Preference for Telehealth Visits. *Journal of General Internal Medicine,* 31**,** 269-275.

PRESTON, J., BROWN, F. W. & HARTLEY, B. 1992. Using telemedicine to improve health care in distant areas. *Psychiatric Services,* 43**,** 25-32.

RCPSYCH. 2020. *Digital - COVID-19 guidance for clinicians* [Online]. London. Available: https://www.rcpsych.ac.uk/about-us/responding-to-covid-19/responding-to-covid-19-guidance-for-clinicians/digital-covid-19-guidance-for-clinicians [Accessed 10/02/2022].

REESE, R. J., MECHAM, M. R., VASILJ, I., LENGERICH, A. J., BROWN, H. M., SIMPSON, N. B. & NEWSOME, B. D. 2016. The effects of telepsychology format on empathic accuracy and the therapeutic alliance: An analogue counselling session. *Counselling and Psychotherapy Research,* 16**,** 256-265.

REINHARDT, I., GOUZOULIS-MAYFRANK, E. & ZIELASEK, J. 2019. Use of Telepsychiatry in Emergency and Crisis Intervention: Current Evidence. *Current Psychiatry Reports,* 21**,** 63.

ROBERTS, N., HU, T., AXAS, N. & REPETTI, L. 2017. Child and adolescent emergency and urgent mental health delivery through telepsychiatry: 12-month prospective study. *Telemedicine and e-Health,* 23**,** 842-846.

ROHLAND, B. M., SALEH, S. S., ROHRER, J. E. & ROMITTI, P. A. 2000. Acceptability of Telepsychiatry to a Rural Population. *Psychiatric Services,* 51**,** 672-674.

RUSKIN, P. E., SILVER-AYLAIAN, M., KLING, M. A., REED, S. A., BRADHAM, D. D., HEBEL, J. R., BARRETT, D., KNOWLES, F., 3RD & HAUSER, P. 2004. Treatment outcomes in depression: comparison of remote treatment through telepsychiatry to in-person treatment. *Am J Psychiatry,* 161**,** 1471-6.

SALES, C. P., MCSWEENEY, L., SALEEM, Y. & KHALIFA, N. 2018. The use of telepsychiatry within forensic practice: a literature review on the use of videolink – a ten-year follow-up. *The Journal of Forensic Psychiatry & Psychology,* 29**,** 387-402.

SCHOPP, L., JOHNSTONE, B. & MERRELL, D. 2000. Telehealth and neuropsychological assessment: New opportunities for psychologists. *Professional Psychology: Research and Practice,* 31**,** 179.

SCHUTTE, J. L., MCCUE, M. P., PARMANTO, B., MCGONIGLE, J., HANDEN, B., LEWIS, A., PULANTARA, I. W. & SAPTONO, A. 2015. Usability and Reliability of a Remotely Administered Adult Autism Assessment, the Autism Diagnostic Observation Schedule (ADOS) Module 4. *Telemedicine and e-Health,* 21**,** 176-184.

SEIDEL, R. W. & KILGUS, M. D. 2014. Agreement between telepsychiatry assessment and face-to-face assessment for emergency department psychiatry patients. *Journal of telemedicine and telecare,* 20**,** 59-62.

SHARMA, G. & DEVAN, K. 2021. The effectiveness of telepsychiatry: thematic review. *BJPsych Bulletin***,** 1-8.

SHORE, J. 2015. The evolution and history of telepsychiatry and its impact on psychiatric care: Current implications for psychiatrists and psychiatric organizations. *Int Rev Psychiatry,* 27**,** 469-75.

SHORE, J. H., SAVIN, D., ORTON, H., BEALS, J. & MANSON, S. M. 2007. Diagnostic Reliability of Telepsychiatry in American Indian Veterans. *American Journal of Psychiatry,* 164**,** 115-118.

SHORE, J. H., SCHNECK, C. D. & MISHKIND, M. C. 2020. Telepsychiatry and the Coronavirus Disease 2019 Pandemic—Current and Future Outcomes of the Rapid Virtualization of Psychiatric Care. *JAMA Psychiatry,* 77**,** 1211-1212.

STRINGER, S., CHURCH, L., KEYNEJAD, R. & HURN, J. 2020. *Psychiatry PR N*, Oxford University Press, USA.

TAYLOR, F. K. 1967. The Role of Phenomenology in Psychiatry. *British Journal of Psychiatry,* 113**,** 765-770.

TRZEPACZ, P. T. & BAKER, R. W. 1993. *The psychiatric mental status examination*, Oxford University Press.

UNÜTZER, J., KIMMEL, R. J. & SNOWDEN, M. 2020. Psychiatry in the age of COVID-19. *World psychiatry : official journal of the World Psychiatric Association (WPA),* 19**,** 130-131.

URNESS, D., HAILEY, D., DELDAY, L., CALLANAN, T. & ORLIK, H. 2004. The status of telepsychiatry services in Canada: a national survey. *J Telemed Telecare,* 10**,** 160-4.

VARKER, T., BRAND, R. M., WARD, J., TERHAAG, S. & PHELPS, A. 2019. Efficacy of synchronous telepsychology interventions for people with anxiety, depression, posttraumatic stress disorder, and adjustment disorder: A rapid evidence assessment. *Psychological services,* 16**,** 621.

VON HAFFTEN, A. 2021. Telepsychiatry Practice Guidelines. Available: https://www.psychiatry.org/psychiatrists/practice/telepsychiatry/toolkit/practice-guidelines [Accessed 10/02/2022].

WITTSON, C. L. & BENSCHOTER, R. 1972. Two-way television: helping the Medical Center reach out. *Am J Psychiatry,* 129**,** 624-7.

WOOTTON, R., YELLOWLEES, P. & MCLAREN, P. 2003. *Telepsychiatry and e-mental health*, Royal Society of Medicine Press London.

ZHAO, L., CHEN, J., LAN, L., DENG, N., LIAO, Y., YUE, L., CHEN, I., WEN, S. W. & XIE, R.-H. 2021. Effectiveness of Telehealth Interventions for Women With Postpartum Depression: Systematic Review and Meta-analysis. *JMIR Mhealth Uhealth,* 9**,** e32544.
